# Noninvasive Tracking of mPEG-poly(Ala) Hydrogel-Embedded MIN6 Cells after Subcutaneous Transplantation in Mice

**DOI:** 10.3390/polym13060885

**Published:** 2021-03-13

**Authors:** Jyuhn-Huarng Juang, Hsiu-Chao Lin, Chen-Yi Chen, Chen-Wei Kao, Chen-Ling Chen, Shu-Ting Wu, Sung-Han Lin, Chia-Rui Shen, Jiun-Jie Wang, Zei-Tsan Tsai, I-Ming Chu

**Affiliations:** 1 Division of Endocrinology and Metabolism, Department of Internal Medicine and Center for Tissue Engineering, Chang Gung Memorial Hospital, Taoyuan 33305, Taiwan; je3474@gmail.com (C.-Y.C.); lian8807111@gmail.com (C.-W.K.); jenny74513@gmail.com (C.-L.C.); 2 Department of Medicine, College of Medicine, Chang Gung University, Taoyuan 33302, Taiwan; 3 Department of Chemical Engineering, National Tsing Hua University, Hsinchu 300044, Taiwan; hsiuchaolin324@gmail.com; 4 Department of Medical Biotechnology and Laboratory Science, College of Medicine, Chang Gung University, Taoyuan 33302, Taiwan; proteinwhite@livemail.tw (S.-T.W.); crshen@mail.cgu.edu.tw (C.-R.S.); 5 Department of Medical Imaging and Radiological Sciences, College of Medicine, Chang Gung University, Taoyuan 33302, Taiwan; image.lin@gmail.com (S.-H.L.); jiunjie.wang@gmail.com (J.-J.W.); 6 Molecular Imaging Center, Chang Gung Memorial Hospital, Taoyuan 33305, Taiwan; zeitsan@ms9.hinet.net

**Keywords:** thermosensitive hydrogels, MIN6 cells, subcutaneous transplantation, magnetic resonance imaging, bioluminescence imaging

## Abstract

Recently, we demonstrated the feasibility of subcutaneous transplantation of MIN6 cells embedded in a scaffold with poly(ethylene glycol) methyl ether (mPEG)-poly(Ala) hydrogels. In this study, we further tracked these grafts using magnetic resonance (MR) and bioluminescence imaging. After being incubated overnight with chitosan-coated superparamagnetic iron oxide (CSPIO) nanoparticles and then mixed with mPEG-poly(Ala) hydrogels, MIN6 cells appeared as dark spots on MR scans. For in vivo experiments, we transfected MIN6 cells with luciferase and/or incubated them overnight with CSPIO overnight; 5 × 10^6^ MIN6 cells embedded in mPEG-poly(Ala) hydrogels were transplanted into the subcutaneous space of each nude mouse. The graft of CSPIO-labeled MIN6 cells was visualized as a distinct hypointense area on MR images located at the implantation site before day 21. However, this area became hyperintense on MR scans for up to 64 days. In addition, positive bioluminescence images were also observed for up to 64 days after transplantation. The histology of removed grafts showed positive insulin and iron staining. These results indicate mPEG-poly(Ala) is a suitable scaffold for β-cell encapsulation and transplantation. Moreover, MR and bioluminescence imaging are useful noninvasive tools for detecting and monitoring mPEG-poly(Ala) hydrogel-embedded MIN6 cells at a subcutaneous site.

## 1. Introduction

Islet transplantation is a promising treatment for patients with diabetes. Currently, the liver is the site of choice for clinical islet transplantation. The major disadvantage of this site is that many grafts are lost or damaged in the early stages of transplantation [1,2]. Additional disadvantages include procedure-associated complications and difficulties in the monitoring and retrieval of islet grafts [3,4,5]. By contrast, islet transplantation into a subcutaneous space offers the advantages of a larger space, a less invasive procedure, and easier graft monitoring and removal. However, it has poor efficacy, possibly due to poor oxygen tension and blood supply and a lack of early neovascularization in subcutaneous tissue [3,6,7].

Biocompatible scaffolds are good for promoting neovascularization and improving islet survival at a subcutaneous site [8]. Thermosensitive polymeric systems that can form semisolid gels through a sol-to-gel transition are particularly useful as cell scaffolds in transplantation. The three-dimensional (3D) networks that characterize these hydrogels bear similarity to the extracellular matrix [9,10,11]. Many polymeric biomaterials are biocompatible and biodegradable and have been used in cell transplantation [12,13,14]. Reverse thermosensitive materials can serve as delivery vehicles for cells or therapeutic agents in biomedical fields [15,16,17,18]. Polypeptide copolymer hydrogels have the advantages of low gelation concentration, distinct architecture, and reversibility [19,20]. Furthermore, the availability of different amino acids enables the design of diverse types of hydrogels to meet specific requirements [21]. In particular, ionic poly-peptide copolymers have the potential to ionically interact with oppositely charged drugs or proteins to achieve extended release [11,22].

The mouse insulinoma-derived MIN6 β-cell line shares several characteristics with pancreatic β-cells, such as insulin secretion in response to glucose stimulation [23]. Sobel et al. have characterized in vivo mouse models for subcutaneous transplantation of MIN6 cells in Matrigel hydrogels and HyStem-C hydrogels [24]. Since the MIN6 cell line’s viability is better for cell clusters than separated cells [25], the poly(ethylene glycol)-poly(peptide) thermosensitive hydrogels are beneficial because they provide spaces for cell expansion and clustering. In addition, they can encapsulate and deliver cells to specific sites with minimal invasiveness. They enable in situ gelling upon being injected into appropriate sites in the body [26]. For instance, we recently demonstrated the feasibility of using poly(ethylene glycol) methyl ether (mPEG)-poly(Ala) hydrogels as delivery carriers for subcutaneous transplantation of MIN6 cells [27].

To better understand the fate of islets after transplantation, accurate, reproducible, and noninvasive imaging is needed. Such approaches have been investigated by using different imaging modalities, such as positron emission tomography (PET), single-photon emission computed tomography (SPECT), magnetic resonance imaging (MRI), ultrasonography (US), bioluminescence imaging (BLI) fluorescence imaging, magnetic particle imaging (MPI), and photoacoustic imaging (PAI) [28,29]. In this study, we subcutaneously transplanted MIN6 cells embedded in a scaffold with mPEG-poly(Ala) hydrogels and examined these grafts for up to 65 days by using MR and bioluminescence imaging. Our results revealed that both tools are useful for detecting and monitoring mPEG-poly(Ala) hydrogel-embedded MIN6 cells at the subcutaneous site.

## 2. Materials and Methods

### 2.1. Materials

mPEG (Mn 2000) and l-alanine were purchased from Sigma-Aldrich (St Louis, MO, USA). Trifluoroacetic acid-d (TFA-d) and trifluoroacetic acid (TFA) were purchased from Alfa Aesar (Wall Hill, MA, USA). Ammonium hydroxide solution (28–30%), diethyl ether, toluene, hexane, and ethanol (95%) were purchased from Echo Chemicals (Toufen, Miaoli, Taiwan). Dimethyl sulfoxide (DMSO) was obtained from J.T. Baker (Center Valley, PA, USA). Dulbecco’s modified eagle’s medium (DMEM), fetal bovine serum (FBS), and antimycotic antibiotics were purchased from Gibco (Grand Island, NY, USA). Tetrahydrofuran (THF) was purchased from TEDIA (Fairfield, OH, USA); dichloromethane (DCM), *N,N*-dimethylformamide (DMF), and chloroform were obtained from AVANTOR (Center Valley, PA, USA) and dried over CaH2 before being used. CSPIO was prepared via a chemical co-precipitation method [30].

### 2.2. Synthesis of l-alanine N-carboxyanhydride (NCA-Ala) and mPEG-poly(Ala) diblock copolymers

The mPEG-poly(Ala) diblock copolymer was prepared via the ROP of l-alanine-*N*-carboxyanhydride (NCA-Ala), with mPEG-NH_2_ serving as the macroinitiator [27]. Briefly, mPEG-NH_2_ was dissolved in 15 mL of toluene, and azeotropic distillation was used to obtain a final volume of approximately 5 mL to remove residual water. A 200 mL solvent containing a mixture of anhydrous chloroform and *N*,*N*-dimethylamide (2:1) and NCA-Ala powder were added to the flask together. The reaction was maintained at 40 °C for 1 day. The product was then precipitated in ice-cold diethyl ether, solubilized in DMSO, and then dialyzed (Molecular weight cut-off 1000 Da) with a spectrum dialysis bag against reverse osmosis water for at least three days. The mPEG-poly(Ala) diblock copolymers were dried through lyophilization and stored under vacuum until further use.

### 2.3. Culture of MIN6 Cells

MIN6 cells were cultured in DMEM supplemented with 1% penicillin streptomycin and 15% heat-inactivated fetal bovine serum (FBS). The MIN6 cells were placed in 10-cm^2^ tissue culture dishes and incubated at 37 °C in the presence of 5% carbon dioxide. Media change was done every 3 days and cells were passaged weekly.

### 2.4. Uptake of Chitosan-Coated Superparamagnetic Iron Oxide (CSPIO) Nanoparticles by MIN6 Cells

MIN6 cells were incubated overnight with CSPIO nanoparticles and then the intracellular iron content was examined by Prussian blue staining. MIN6 cells were washed with PBS to remove excess iron particles and then fixed in 4 vol% formaldehyde solution for 30 min. After fixation, the cells were stained for the presence of intracellular iron with freshly prepared potassium ferrocyanate solution (mixture of equal volume of 4 wt% potassium ferrocyanate with 4 vol% hydrochloric acid) for 30 min. After washing with distilled water, the cells were examined using a microscope to determine the labeling efficiency. Cells with intracellular blue particles were considered labeled. 

### 2.5. In vitro MR Scanning

MR imaging was performed on a 7.0 T MRI system (Bruker, Ettlingen, Germany). 1 × 10^6^ MIN6 cells were incubated overnight with CSPIO nanoparticles for 24 h at 37 °C and washed three times in PBS. All samples were scanned by using a fast gradient recalled echo pulse sequence (repetition time (TR)/echo time (TE) = 3000 msec/70 msec). The percentage contrast enhancement (%) was calculated by the following equation: percentage of enhancement (%) = (SIpost − SIpre)/SIpre × 100, where SIpost is the signal intensity measured from the phantom of cells in cells treated with the contrast agent, CSPIO; and SIpre is the signal intensity for cells alone.

### 2.6. In vivo Transplantation and Imaging

The animal experiments were approved by the institutional animal ethics committee. Animals were purchased from the National Laboratory Animal Center, Taipei, Taiwan. Nude mice aged 8–12 weeks were used as recipients of transplantation. For in vivo imaging, we transfected MIN6 cells with luciferase and/or incubated them overnight with CSPIO before transplantation; 5 × 10^6^ MIN6 cells embedded in the 100 μL hydrogel solution were injected into the subcutaneous space of each mouse. After transplantation, serial MR and bioluminescence images were acquired in 6 and 4 recipients, respectively. MR Images were acquired on a 7.0 T MRI system (Bruker, Ettlingen, Germany) using a home-made surface coil with the following parameters: slice thickness = 0.5 mm, TR = 3700 msec, TE = 37 msec. A T2 weighted gradient-recalled echo sequence was acquired for all subjects. Bioluminescence imaging was performed by in vivo imaging system (IVIS, Xenogen, PerkinElmer, Inc., Waltham, MA USA).

### 2.7. Histological Study of Grafts of Hydrogel-Embedded MIN6 Cells

Grafts of hydrogel-embedded MIN6 cells were removed up to 65 days after transplantation. They were fixed in a formalin solution and processed for paraffin embedding and sectioning. Sections of grafts were stained with hematoxylin and eosin, for iron with Prussian blue, and for endocrine β-cells with a guinea pig anti-swine insulin antibody (Dako, CA, USA).

## 3. Results and Discussion

### 3.1. Uptake of CSPIO Nanoparticles by MIN6 Cells

**Figure 1:**
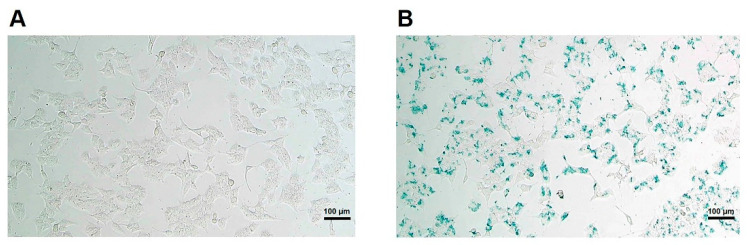
Uptake of chitosan-coated superparamagnetic iron oxide (CSPIO) nanoparticles by MIN6 cells. MIN6 cells were incubated overnight without (**A**) or with (**B**) CSPIO nanoparticles. The intracellular iron content was examined by Prussian blue staining.

To track MIN6 cells in vivo by MRI, it is essential to demonstrate the cellular uptake of the contrast agent with positive images of MR scans. CSPIO nanoparticles are one kind of MR contrast agent [30] which does not affect islet viability and insulin secretion [31], and they have been used for long-term tracking of islet isografts [31,32] and allografts [31,33]. To examine the cellular uptake of CSPIO nanoparticles, MIN6 cells were incubated overnight with CSPIO nanoparticles and then the intracellular iron content was examined by Prussian blue staining. Figure 1A shows the absence of blue stain in the MIN6 cells without CSPIO loading. In contrast, the blue spots were located in the cytoplasms of some CSPIO-loaded MIN6 cells (Figure 1B), indicating CSPIO nanoparticles were taken up by these cells. These findings are consistent with our previous observation that CSPIO could be introduced into cells, including two pancreatic β-cell lines, NIT-1 and β-TC6 [34].

### 3.2. In vitro MR Image of MIN6 Cells

**Figure 2:**
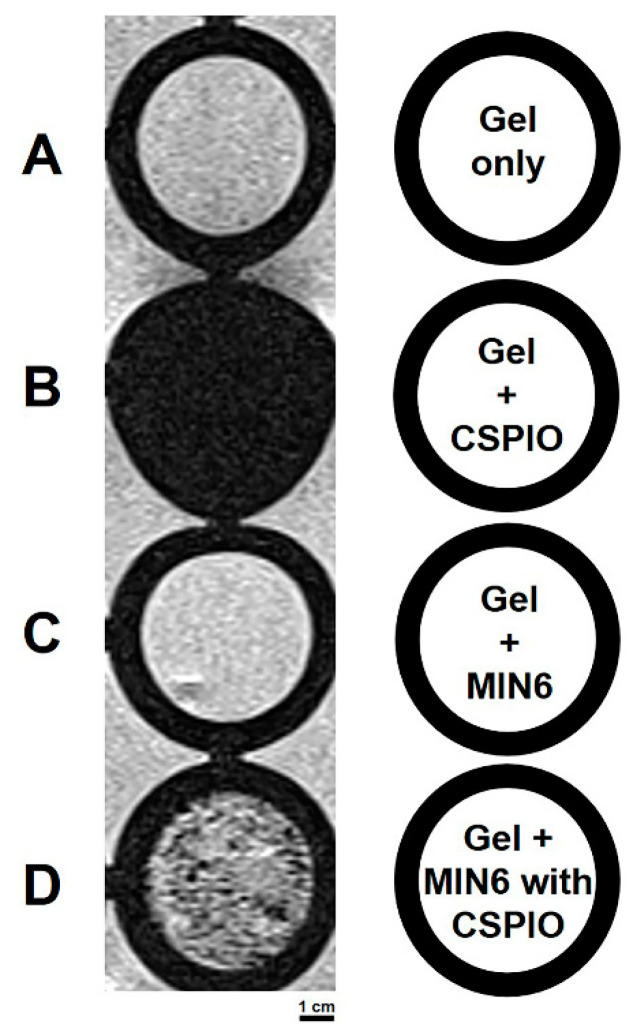
In vitro MRI of mPEG-poly(Ala) hydrogels, hydrogels with CSPIO nanoparticles, and MIN6 cells mixed with hydrogels after incubating with or without chitosan-coated superparamagnetic iron oxide (CSPIO) nanoparticles. All were scanned by a 7.0 T MRI system. Gel only (**A**) was used as a negative control and Gel+CSPIO (**B**) was used as a positive control. In contrast to unlabeled MIN6 cells (**C**), CSPIO-loaded MIN6 cells (**D**) appeared as dark spots.

We then perform in vitro MRI on mPEG-poly(Ala) hydrogels (a negative control), hydrogels with CSPIO nanoparticles (a positive control), and MIN6 cells mixed with hydrogels after incubating with or without CSPIO nanoparticles (Figure 2). As expected, there was a background image in hydrogels (Figure 2A) and a complete dark image in hydrogels with CSPIO nanoparticles (Figure 2B). In contrast to a background image in MIN6 cells without CSPIO labeling (Figure 2C), CSPIO-loaded MIN6 cells appeared as dark spots (Figure 2D), corresponding to the locations of loaded cells [34]. Visualization of CSPIO-labeled MIN6 cells by in vitro MRI was fundamental for our following in vivo detection of CSPIO-labeled MIN6 cells after transplantation. 

### 3.3. In Vivo MR Images of Hydrogel-Embedded MIN6 Cells after Subcutaneous Transplantation

**Figure 3:**
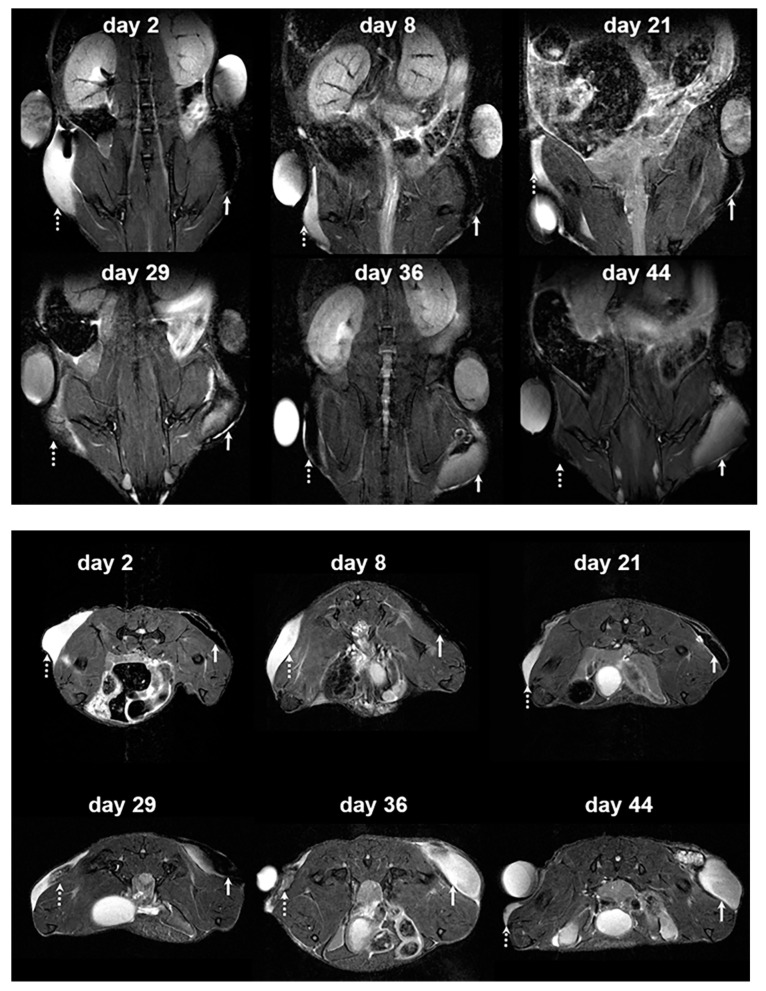
In vivo MR image of hydrogel and hydrogel-embedded MIN6 cells after subcutaneous transplantation. 5 × 10^6^ CSPIO-labeled MIN6 cells embedded in mPEG-poly(Ala) hydrogels were subcutaneously transplanted into the right flank of a nude mouse. The hydrogel without MIN6 cells was transplanted into the left flank as a negative control. The recipient was scanned by a 7.0 T MRI system with coronal (upper panel) and transverse (lower panel) sections. The graft of CSPIO-labeled MIN6 cells was indicated by solid arrows and the hydrogel without MIN6 cells was indicated by dashed arrows.

For in vivo MRI, we transplanted 5×10^6^ CSPIO-labeled MIN6 cells embedded in mPEG-poly(Ala) hydrogels into the subcutaneous tissue of each nude mouse. Six recipients were scanned by a 7.0 T MRI system for up to 64 days. As shown in Figure 3, the graft of CSPIO-labeled MIN6 cells on the right flank (indicated by solid arrows) was visualized as a distinct hypointense area on MR images located at the implantation site between day 2-21. Then, it became hyperintense on MR scans between 29 and 44 days. In contrast, the implanted hydrogel on the left flank (dashed arrows in Figure 3) appeared hyperintense on T2 weighted MR images, which gradually decreased in size over time. As we previously visualized CSPIO-labeled islets under mouse kidney capsules on MR scans as persistent hypointense areas after syngeneic and allogeneic transplantation [31,32,33], CSPIO-laden MIN6 cell grafts were expected to show hypointense areas on MR images early after transplantation. After 29 days, the graft became hyperintense on MR scans because insulinoma-derived MIN6 cells proliferated to generate daughter cells which did not contain CSPIO nanoparticles. Meanwhile, the hyperintense MR images of the implanted hydrogels without MIN6 cells gradually declined as a result of degradation of the materials [27]. Although subcutaneous transplantation of MIN6 cells in mice has been investigated [24,35], to the best of our knowledge, we are the first to use MRI for the graft detection and monitoring.

### 3.4. In Vivo Bioluminescence Images of Hydrogel-Embedded MIN6 Cells after Subcutaneous Transplantation

**Figure 4:**
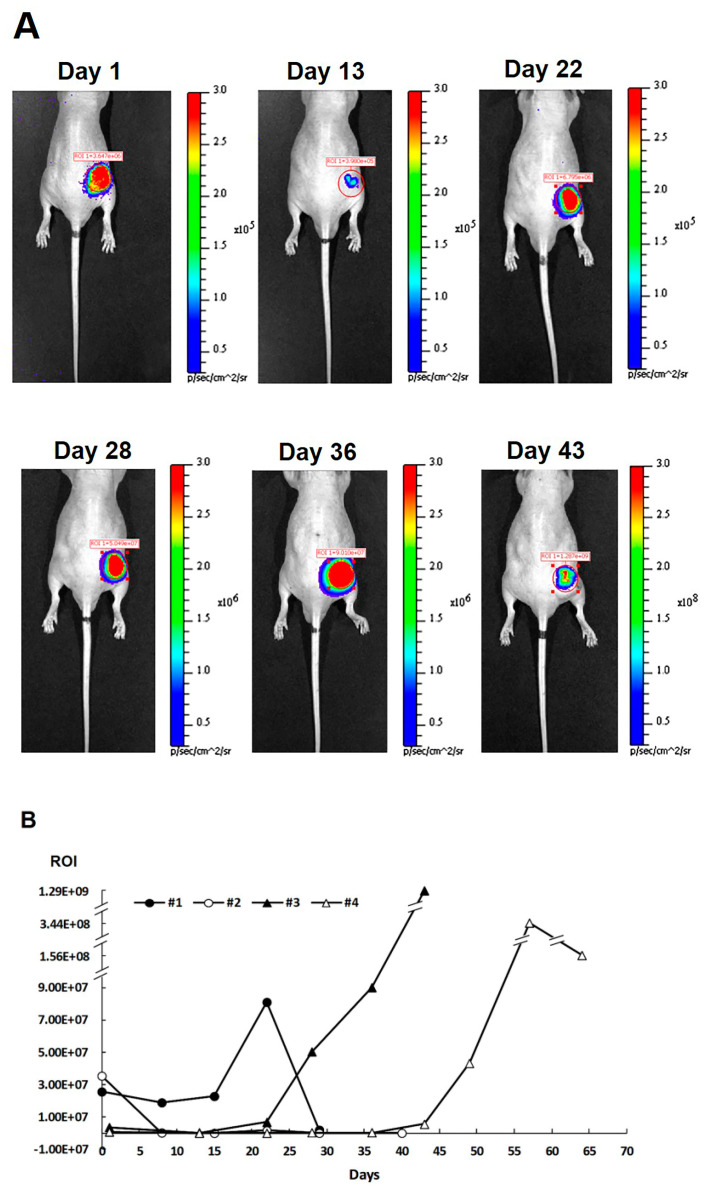
In vivo bioluminescence images of hydrogel and hydrogel-embedded MIN6 cells after subcutaneous transplantation; 5 × 10^6^ luciferase-transfected MIN6 cells embedded in mPEG-poly(Ala) hydrogels were subcutaneously transplanted into the right flank of a nude mouse. The hydrogel without MIN6 cells was transplanted into the left flank as a negative control. Recipients were imaged by in vivo imaging system (IVIS). (**A**) The bioluminescence image of a representative recipient. (**B**) The signal intensities (rigion-of-interest, ROI) of bioluminescence images for all recipients.

Recently, we have applied bioluminescence images in tracking Matrigel-embedded MIN6 cells for 2 weeks after subcutaneous transplantation [35]. In this study, we subcutaneously transplanted 5 × 10^6^ luciferase-transfected MIN6 cells embedded in mPEG-poly(Ala) hydrogels into each nude mouse and then imaged four recipients with IVIS for up to 64 days. Figure 4A shows positive bioluminescence images of single representative recipients at 1, 13, 22, 28, 36, and 43 days, indicating the existence of surviving MIN6 cells after transplantation. However, the signal was weaker at 13 and 43 days compared to other days. The signal intensity decreased at 13 days, possibly due to loss of MIN6 cells [36], and subsequently increased at 22 days owing to tumor growth of MIN6 cells [24]. Regarding the durability of in vivo bioluminescence, we observed a signal drop or loss on days 8, 29, 43, and 64 in four recipients (Figure 4B). The duration of positive bioluminescence images is consistent with reports of subcutaneous hair stem cells (21 days) [37] and intramyocardial or intramuscular human embryonic stem cells (up to 8 weeks) [38]. Taking together the results of MR and bioluminescent images, we demonstrated the graft of mPEG-poly(Ala) hydrogel-embedded MIN6 cells could be tracked at the subcutaneous site for a long period.

### 3.5. Subcutaneous Grafts of Hydrogel-Embedded MIN6 Cells

**Figure 5:**
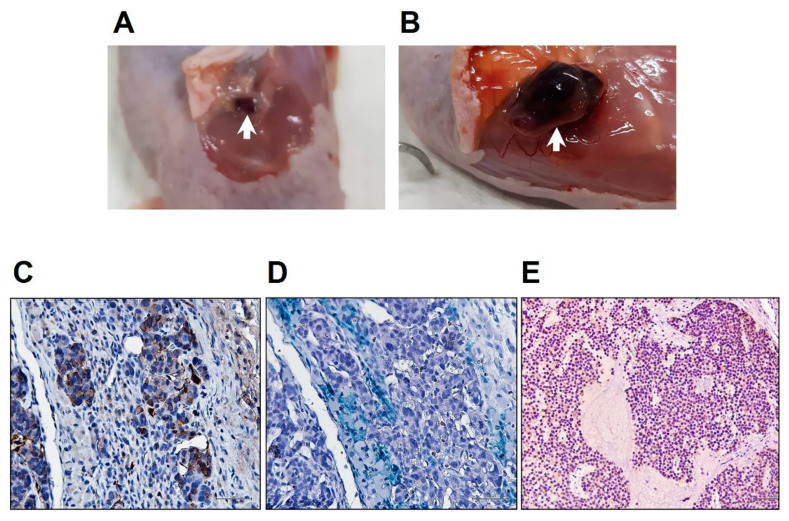
Subcutaneous grafts (indicated by arrows) of hydrogels without (**A**, 0.3 × 0.2 × 0.1 cm) or with (**B**, 1.3 × 1 × 0.6 cm) MIN6 cells at 65 days after transplantation. The latter graft was stained with insulin (**C**, brown) and Prussian blue (**D**, blue). (**E**) Insulin staining (brown) of the MIN6 cell graft at 48 days after transplantation.

Subcutaneous grafts of hydrogels with or without MIN6 cells were removed from recipients up to 65 days after transplantation. The former (Figure 5B, 0.3 × 0.2 × 0.1 cm) were much bigger than the latter (Figure 5A, 1.3 × 1 × 0.6 cm), although both were well vascularized at day 65. Since the mPEG-poly(Ala) hydrogels are biodegradable [27], their graft size decreased with time. This also explains the gradual decline of hyperintense MR images of the implanted hydrogels (Figure 3). In contrast, the grafts of hydrogels with MIN6 cells gradually enlarged because of cell proliferation. To investigate the grafts microscopically, we used an insulin antibody to stain MIN6 cells, Prussian blue to stain iron, and a CD31 antibody to stain endothelial cells. As shown in Figure 5, the graft was positive for insulin (Figure 5D), iron (Figure 5E), and CD31. However, compared to 2-week grafts [27], the hydrogel was barely seen. In addition, MIN6 cells were arranged into clusters and confluence. The MIN6 cell graft at 48 days showed diffuse insulin staining of almost all cells (Figure 5E), indicating tumor growth of MIN6 cells [24]. These results indicate transplanted CSPIO-labeled MIN6 cells in the presence of neovascularization not only survived, but also proliferated at the subcutaneous site.

## 4. Conclusions

We recently demonstrated the feasibility of subcutaneous transplantation of MIN6 cells embedded in a scaffold with mPEG-poly(Ala) hydrogels. In this study, we further tracked these grafts after transplantation. We found CSPIO-labeled and luciferase-transfected MIN6 cells embedded in hydrogels could be detected by MR and bioluminescence imaging, respectively. These results indicate the mPEG-poly(Ala) hydrogel is a suitable scaffold for β-cell encapsulation and transplantation. Moreover, both MR and bioluminescence imaging are useful tools for detecting and monitoring mPEG-poly(Ala) hydrogel-embedded MIN6 cells at the subcutaneous site.

## Data Availability

Not applicable.
